# Predictors of smoking among Swedish adolescents

**DOI:** 10.1186/1471-2458-14-1296

**Published:** 2014-12-17

**Authors:** Junia Joffer, Gunilla Burell, Erik Bergström, Hans Stenlund, Linda Sjörs, Lars Jerdén

**Affiliations:** Department of Public Health and Clinical Medicine, Epidemiology and Global Health, Umeå University, SE-901 87 Umeå, Sweden; Center for Clinical Research Dalarna-Uppsala University, Nissers väg 3, SE-791 82 Falun, Sweden; Department of Public Health and Caring Sciences, Uppsala University, Box 564, SE-751 22 Uppsala, Sweden; Department of Clinical Sciences, Pediatrics, Umeå University, SE-901 87 Umeå, Sweden

**Keywords:** Smoking, Adolescence, Smokeless tobacco, Snus, Self-esteem, Attitudes, Longitudinal study

## Abstract

**Background:**

Smoking most often starts in adolescence, implying that understanding of predicting factors for smoking initiation during this time period is essential for successful smoking prevention. The aim of this study was to examine predicting factors in early adolescence for smoking in late adolescence.

**Methods:**

Longitudinal cohort study, involving 649 Swedish adolescents from lower secondary school (12–13 years old) to upper secondary school (17–18 years old). Tobacco habits, behavioural, intra- and interpersonal factors and socio-demographic variables were assessed through questionnaires. Descriptive statistics, univariable and multivariable logistic regression were used to identify predicting factors.

**Results:**

Smoking prevalence increased from 3.3% among 12–13 year olds to 25.1% among 17–18 year olds. Possible predictors of smoking were: female sex, lower parental education, poorer family mood, poorer self-rated health, poorer self-esteem, less negative attitude towards smoking, binge drinking, snus use and smoking. In a multivariable logistic regression analysis, female sex (OR 1.64, CI 1.08-2.49), medium and low self-esteem (medium: OR 1.57, CI 1.03-2.38, low: 2.79, CI 1.46-5.33), less negative attitude towards smoking (OR 2.81, CI 1.70-4.66) and ever using snus (OR 3.43, CI 1.78-6.62) remained significant independent predicting factors.

**Conclusions:**

The study stresses the importance of strengthening adolescents’ self-esteem, promoting anti-smoking attitudes in early adolescence, as well as avoidance of early initiation of snus. Such measures should be joint efforts involving parents, schools, youth associations, and legislating authorities.

## Background

Tobacco consumption is a global pandemic, killing half of all lifetime users. In 2011, six million people died as a result of tobacco use, and by the year 2030 eight million people is expected to die annually [1]. According to the WHO study Health Behaviour in School-aged Children (HBSC), approximately 18% of 15 year old adolescents smoke cigarettes at least once a week, corresponding figures in Sweden are 15% among girls and 13% among boys [2]. Nicotine addiction is usually developed during adolescence and has been referred to as a ‘paediatric disease’ [3]. After the age of twenty, the risk of starting to smoke on a regular basis decreases [4], suggesting that if adolescents can be kept tobacco free, most of them will never start [5].

### Socio-demographic factors

The HBSC study shows complex socio-demographic and culturally related gender patterns of smoking. At age 15, boys generally report more smoking (weekly) than girls, but in some high-income countries, however, girls smoke more than boys [2]. Among adults in high income countries, e.g. Great Britain, smoking follows a steep social class gradient with more smoking in lower social classes [6]. In youth, findings are conflicting and difficult to interpret, partly due to differences in measures of socioeconomic status [7]. A U.S. longitudinal study found that parents’ educational level was associated with current smoking among adolescents, but did not predict smoking initiation at a one-year follow-up [8]. Results from a U.S. prospective birth cohort indicated that low childhood socioeconomic status increased the risk of both smoking initiation and progression to regular smoking [9].

### Interpersonal factors

Among factors within the individual’s sphere (interpersonal factors) smoking among friends [10] and in the family are well known predictors [11]. Young people become smokers in a social context. Individual and contextual factors are intertwined in a complex interaction in the process from initiation to maintenance [12]. Family connectedness seems to play a key role in protecting adolescents from smoking [13]. It has been claimed that social factors are more important for smoking initiation while individual factors are more important for persistence [14].

### Intrapersonal factors

Longitudinal findings from a U.S. cohort found subjective poor health to be a significant predictor of transition into smoking among girls [13]. Predictive associations between self-esteem and smoking have been regarded as inconclusive. Short term longitudinal findings among adolescents in the Netherlands indicated that low self-esteem was related to smoking onset among girls [15]. Among young New Zealanders, no predictive association between self-esteem and smoking was found [16]. It is debated if attitudes towards smoking predict smoking initiation, with some studies showing associations [17], while others do not [18], suggesting a need for further studies [18].

### Behavioural factors

Previous research has demonstrated that both tobacco and alcohol use seem to predict future smoking [10]. Whether smokeless tobacco predicts smoking is however not clear. A study of young people in Norway shows that the odds of being a lifetime smoker is higher among those who start using snus (moist snuff) before the age of 16 [19]. One U.S. prospective study shows that use of smokeless tobacco at baseline facilitated smoking at follow-up [20], while another U.S. study showed the opposite [21]. Another Norwegian study suggests that snus use predicts a mixed use of snus and smoking, not smoking solely [22]. In contrast to risk behaviours, physical activity (participation in sports) seems to protect against both smoking and snus use [23].

### Sweden

The overall use of cigarettes in Sweden has continually declined over the last decades. A specific characteristic of Sweden is the high use of moist smokeless tobacco, ‘snus’ [24]. According to the Swedish part of the HBSC-study, 7% of the boys and 2% of the girls in the age of 15 report snus use at least once a week [25]. Combined smoking and snus use is more common among boys. A Swedish study following adolescents from 11 to 18 years of age demonstrated that initiation of snus and cigarettes close in time predicted future smoking [26].

Thus, multiple factors seem to predict smoking in adolescence, but the findings are not conclusive. We still need more information about the development of smoking behaviour in early adolescence, information that is imperative for effective preventive interventions [27]. To fully understand the complex phenomena of risk behaviours, i.e. smoking, research questions need to be addressed with longitudinal studies during the developmental phase of adolescence [28]. Hence, the aim of this study was to examine predicting factors in early adolescence for smoking in late adolescence.

## Methods

### Study population and procedures

**Figure 1:**
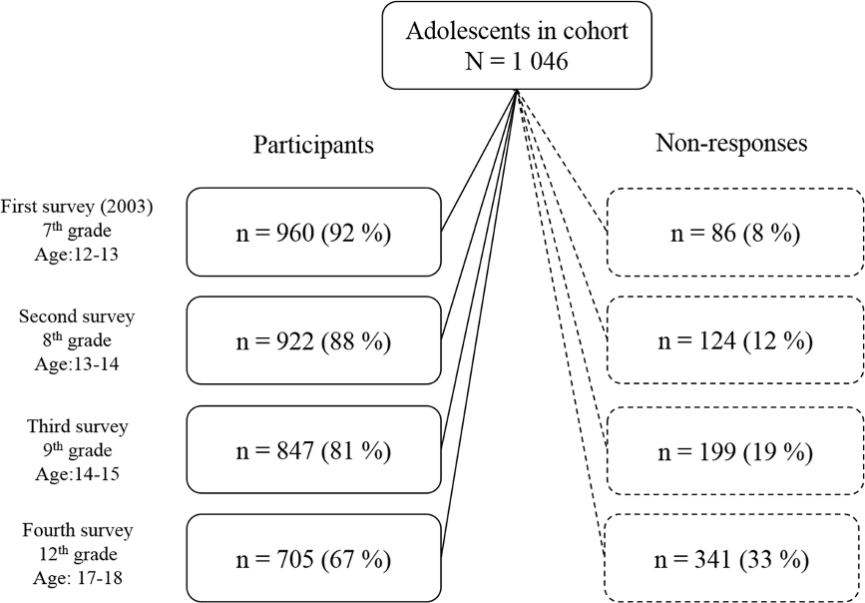
**Participants and non-responses (drop-out and un-identified) in each survey in the cohort study.**

In the study, 1 046 adolescents from three Swedish municipalities, Borlänge, Falun (central Sweden) and Umeå (northern Sweden), were invited to answer a health questionnaire. Seven schools covering both high and low educational level of parents were invited to participate. Data on educational level of parents was obtained from Statistics Sweden, the official national statistical database. All schools accepted the invitation. Informed consent was obtained by a letter sent home to the participants. The consent procedure followed that of ‘opt out’, implying that no active consent was asked for. No parents refused their child to participate, thus all 1 046 invited adolescents are considered as the study cohort. The questionnaire was anonymous with a code number. Four surveys have been performed (Figure 1) and this particular study uses data from the first and the fourth survey, which were conducted in 2003 and in 2008. The first survey was made in 7th grade (age 12–13) when the students had left primary school and entered lower secondary school. The second survey was made in 8th grade and the third in 9th grade. Teachers administrated the questionnaires during class hours. The fourth survey was made the last school year of upper secondary school, in 12th grade (age 17–18). For logistic reasons, since Swedish adolescents change schools and classes split from grade 9 (age of 15–16), this last survey was made using a postal questionnaire.

### Participants and non-responses

Of the 1 046 adolescents in the cohort, 984 participated in the first survey and of these 649 participated also in the fourth survey. In 7th grade 24 adolescents where un-identified as they removed the code number from the questionnaire. In this analysis, they are referred to as ‘non-responses’. Corresponding numbers in 8th, 9th and 12th grade were 68, 59 and 20 respondents respectively. When comparing participants and non-participants at baseline no significant differences regarding age or gender were found. When comparing background characteristics between participants at baseline and follow-up to those lost to follow-up, a significantly higher proportion of boys, respondents born outside Sweden and snus users were lost to follow-up (Table 1).

**Table 1:** **Baseline characteristics among participants at baseline and follow-up, and those lost to follow-up**

	Participants at baseline and follow-up	Lost to follow-up	Chi ^2^
	n (%)	n (%)	***P***
**Gender**			
**Boys**	292 (45.0)	187 (60.1)	
**Girls**	357 (55.0)	124 (39.9)	<0.01
**Age**			
**12 years old**	154 (23.8)	57 (18.4)	
**13 years old**	475 (73.5)	239 (77.3)	
**14 years old**	17 (2.6)	13 (4.2)	0.09
**Country of birth**			
**Born in Sweden**	612 (94.4)	282 (91.0)	
**Born outside Sweden**	36 (5.6)	28 (9.0)	0.04
**Residence**			
**City/Town**	350 (55.6)	180 (60.8)	
**Village**	160 (25.4)	64 (21.6)	
**Rural area**	120 (19.0)	52 (17.6)	0.30
**Parental education**			
**High**	352 (54.7)	146 (47.9)	
**Low**	292 (45.3)	159 (52.1)	0.05
**Mood in family**			
**Good**	572 (90.1)	270 (89.1)	
**Not good**	63 (9.9)	33 (10.9)	0.65
**Self-rated health**			
**High**	333 (51.7)	156 (50.5)	
**Medium**	272 (42.2)	122 (39.5)	
**Low**	39 (6.1)	31 (10.0)	0.08
**Self-esteem**			
**High**	362 (56.7)	194 (63.6)	
**Medium**	223 (35.0)	86 (28.2)	
**Low**	53 (8.3)	25 (8.2)	0.10
**Attitudes towards smoking**			
**Very negative**	554 (86.2)	252 (81.8)	
**Not very negative**	89 (13.8)	56 (18.2)	0.08
**Physical exercise**			
**High**	480 (77.4)	226 (75.6)	
**Low**	140 (22.6)	73 (24.4)	0.54
**Binge drinking**			
**No**	595 (93.4)	274 (91.0)	
**Once or more**	42 (6.6)	27 (9.0)	0.19
**Snus use**			
**No**	588 (91.9)	260 (86.4)	
**Previous or current use**	52 (8.1)	41 (13.6)	0.01
**Smoking**			
**No**	618 (96.7)	286 (94.4)	
**Yes**	21 (3.3)	17 (5.6)	0.09

### Questionnaire

A questionnaire focusing on self-rated health, health- and risk behaviours, empowerment, attitudes and socio-demographic characteristics was developed. Most questions derived from the Swedish part of the HBSC survey [29] and from established Swedish surveys [30, 31]. The questionnaire was tested for reliability through test-retest that was performed during a pilot study to the cohort study, items with kappa values below 0,40 were excluded [32].

#### Socio-demographic variables

Country of birth was assessed by the question ‘In which country were you born?’ and respondents were dichotomized as born in- or outside Sweden. Residence was self-reported with three alternatives, city/town, village and rural area. Socioeconomic status (SES) was assessed by parental educational level and data was obtained from Statistics Sweden through data linkage on an individual level. Families in which at least one of the parents had a college or university degree were defined as high SES.

#### Interpersonal variable

The variable ‘perceived mood in the family’ [33] was measured by the question ‘How do you consider the mood in your family?’ with a 5-grade ordinal scale. The answers ‘very good’ and ‘rather good’ were characterized as ‘good’. Family was defined as ‘the persons you live together with’.

#### Intrapersonal variables

Self-rated health was measured by the question ‘How do you feel most of the time?’, using a 5-grade ordinal scale. In the analyses, ‘high’ was defined by the answer ‘very good’, ‘medium’ by the answer ‘rather good’, and ‘low’ by the answers ‘neither good, nor bad’, ‘rather bad’, or ‘very bad’. Self-esteem was measured by the question ‘Do you like yourself?’ with a 3-grade ordinal scale [31]. ‘Yes, most often’ was defined as ‘high’, ‘yes, sometimes’ as ‘medium’ and ‘no, seldom’ as ‘low’. Health attitudes were measured by asking which factors adolescents found important to stay healthy. One of the issues to evaluate on a 4-grade ordinal scale was ‘not to smoke’. The answers were dichotomized with those who considered it ‘very important’ as one group and the rest of the answers as another.

#### Behavioural variables

Physical exercise was measured by a 7-grade ordinal scale with the question ‘How often do you usually exercise in your spare time (i.e. outside school) so you become breathless or sweating?’ [29]. An answer of ‘two or three times weekly’ or ‘more often’ was regarded as ‘high’. Binge drinking was assessed by the question ‘Have you ever consumed so much alcohol that you have become really drunk?’ [29], with a 4-grade ordinal scale. The answers were dichotomized in those who had ever been drunk and those who had not. Snus use was assessed by the question ‘Have you ever used snus?’ with a 4-grade ordinal scale ‘no, I have never used snus’, ‘previously but no longer’, ‘sometimes’ and ‘every day’. The last three alternatives were categorized as ‘previous or current use’. Smoking status was measured by the question ‘How often do you smoke these days?’ from the HBSC-study [29] with a 4-grade ordinal scale with the answer alternatives: ‘every day’, ‘at least one time per week but not every day’, ‘less than one time per week’ and ‘I don’t smoke’. In the analyses, a ‘smoker’ included all stages of smoking. Smoking status was both used as a predictor in 7th grade and as the dependent variable in 12th grade.

### Statistics

Data were analysed using SPSS 20.0. Differences in frequency distributions were evaluated by chi^2^-test with p < 0.05 as significance level. Odds ratio (OR) and 95% confidence intervals (CI) were estimated using univariable and multivariable binary logistic regression analysis with a stepwise backward procedure, followed by a stepwise forward procedure. In the multivariable analysis theoretically potential predictors at baseline related to smoking at follow-up were included.

### Ethics

The study was approved by the Research Ethics Committee of the Medical Faculty, Umeå University, reference number 03–073.

## Results

**Figure 2:**
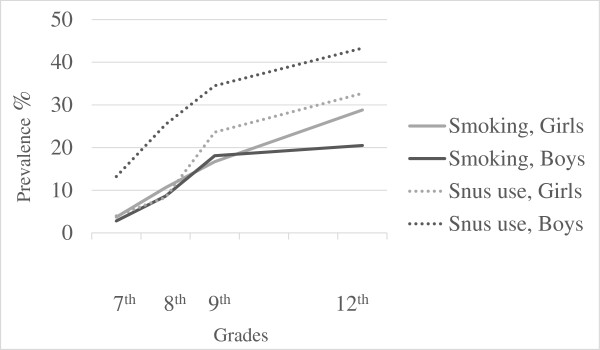
**Prevalence of smoking (daily, weekly, less than weekly), and snus use (daily, occasionally, previous) (n = 649).**

At baseline (7th grade) the mean age of participants was 12.8 years. The prevalence of smoking was 3.3%, increasing to 25.1% at follow-up five years later. Further, 8.1% reported ever using snus, increasing to 37.5% at follow-up. Boys reported higher snus use than girls, and also higher use compared to their own smoking. Figure 2 presents the development of smoking and snus use by gender. At baseline, 2.8% of the boys and 1.4% of the girls reported combined smoking and ever using snus, increasing to 17.2% of the boys and 19.1% of the girls at follow-up. Univariable logistic regression analysis (Table 2) showed several possible significant predictors of smoking at follow-up: female sex, lower parental education, poorer family mood, poorer self-rated health, poorer self-esteem, less negative attitude towards smoking, binge drinking, snus use and smoking. In a multivariable logistic regression analysis (Table 2) four predictors remained independently related to smoking: female sex, poorer self-esteem, less negative attitude towards smoking and ever using snus.

**Table 2:** **Possible predicting factors in 7th grade (12–13 year olds) for smoking in 12th grade (17–18 year olds)**

	Non-smokers 12th grade	Smokers 12th grade	Univariable logistic regression 12th grade		Multivariable logistic regression 12th grade (n = 564)	
Factors 7th grade	n (%)	n (%)	OR	CI	OR	CI
**Gender (n = 646)**						
**Boys**	232 (80)	60 (20)	1.00	Ref	1.00	Ref
**Girls**	252 (71)	102 (29)	1.56	1.09-2.26	1.64	1.08-2.49
**Age (n = 643)**						
**12 years old**	122 (80)	30 (20)	1.00	Ref		
**13 years old**	345 (73)	129 (27)	1.52	0.97-2.38		
**14 years old**	15 (88)	2 (12)	0.54	0.12-2.50		
**Country of birth (n = 645)**						
**Born in Sweden**	453 (74)	156 (26)	1.00	Ref		
**Born outside Sweden**	30 (83)	6 (17)	0.58	0.24-1.42		
**Residence (n = 627)**						
**City/Town**	263 (75)	87 (25)	1.00	Ref		
**Village**	122 (78)	35 (22)	0.87	0.56-1.36		
**Rural area**	86 (72)	34 (28)	1.20	0.75-1.90		
**Parental education (n = 641)**						
**High**	277 (79)	73 (21)	1.00	Ref		
**Low**	203 (70)	88 (30)	1.64	1.15-2.36		
**Mood in family (n = 632)**						
**Good**	436 (77)	133 (23)	1.00	Ref		
**Not good**	36 (57)	27 (43)	2.46	1.44-4.20		
**Self-rated health (n = 641)**						
**High**	257 (78)	74 (22)	1.00	Ref		
**Medium**	203 (75)	68 (25)	1.16	0.80-1.70		
**Low**	20 (51)	19 (49)	3.30	1.67-6.51		
**Self-esteem (n = 635)**						
**High**	293 (81)	69 (19)	1.00	Ref	1.00	Ref
**Medium**	154 (70)	66 (30)	1.82	1.23-2.69	1.57	1.03-2.38
**Low**	28 (53)	25 (47)	3.79	2.08-6.91	2.79	1.46-5.33
**Attitudes towards smoking (n = 640)**						
**Very negative**	433 (78)	119 (22)	1.00	Ref	1.00	Ref
**Not very negative**	46 (52)	42 (48)	3.32	2.09-5.29	2.81	1.70-4.66
**Physical exercise (n = 617)**						
**High**	360 (75)	118 (25)	1.00	Ref		
**Low**	98 (70)	41 (30)	1.28	0.84-1.94		
**Binge drinking (n = 634)**						
**Never**	456 (77)	136 (23)	1.00	Ref		
**Once or more**	20 (48)	22 (52)	3.69	1.95-6.96		
**Snus use (n = 637)**						
**No**	452 (77)	133 (23)	1.00	Ref	1.00	Ref
**Previous or current use**	24 (46)	28 (54)	3.96	2.22-7.07	3.43	1.78-6.62
**Smoking (n = 636)**						
**No**	473 (77)	142 (23)	1.00	Ref		
**Yes**	6 (29)	15 (71)	8.33	3.17-21.86		

## Discussion

The study confirms previous findings of a multi-factorial explanation to initiation and persistence of smoking. Health attitudes, family and intrapersonal factors, binge drinking, smoking and ever using snus all predicted future smoking.

The results showed that poor self-esteem predicted future smoking. As being concluded by McGee and Williams (2000) longitudinal studies addressing this topic are few and the findings are inconclusive. Self-esteem is a concept closely related to self-efficacy, which according to Bandura (1995) is central in models attempting to explain addictive behaviours [34]. In a short-term longitudinal perspective, Engels and colleagues (2005) confirms that self-esteem in the early years of adolescence is linked to smoking in subsequent years among girls [15].

The Theory of Planned Behaviour suggests that behaviours can be predicted by a person’s intention to enact the behaviour. Since attitudes and intentions are closely related, our finding that less strong anti-smoking attitude increases the risk of smoking initiation is in line with this theory. The follow-up time in our study was relatively long; anti-smoking attitudes had a strong association with smoking five years later. Thus, our findings suggest that attitudes towards smoking in early adolescence are related to behaviour later in life.

Initial health attitudes were measured when the adolescents were entering teenage, and only a month earlier had moved to a school level that among adolescents in Sweden is associated with teenage values and growing independence. Therefore, at the time of the first questionnaire, the study cohort may not have fully adopted ‘teenage norms’, i.e. they were probably still much influenced by norms of significant adults, e.g. their parents. We were unable to study the impact of parental smoking since this information was unavailable. It is, however, well documented that parental smoking is a risk factor for adolescent smoking. In a Swedish study with focus group interviews of adolescents, one central theme was that concerned adults make a difference [12].

In this cohort, snus use in early adolescence was a strong predictor for smoking in late adolescence. However, a large drop-out of snus users between baseline and follow-up adds some uncertainty to this finding. The results are consistent with previous findings that use of smokeless tobacco facilitated smoking at follow-up among high school students in California [20], and partly consistent with findings from a Norwegian cohort study of young men in which snus use at baseline elevated the risk of a combined use of snus and smoking at follow-up [22]. Thus, in both studies snus facilitated smoking. The Norwegian study also found that a combined use at baseline implied a higher risk for combined use (daily smoking and daily snus use) at follow-up. As all boys who reported smoking at baseline in our study also reported ever using snus, this indicates a widespread combined use. However, due to small numbers of smokers and snus users at baseline, it is not feasible to divide tobacco use into smokers, snus users and combined users as the groups get too small for proper analyses.

Use of smokeless tobacco varies around the world, but in a number of countries it counts for a substantial part of the total tobacco consumption. In Sweden, health authorities regard snus a less serious health hazard than cigarette smoking [35]. Based on our results, the most important health hazard of snus use could be the early introduction of nicotine and by that the risk of nicotine addiction. A recent Swedish study found symptoms of nicotine dependence to be more common among adolescents who used both cigarettes and snus [36]. An implication should be that preventive efforts against snus should be introduced in an early age.

The higher risk for girls to become smokers, prominent in many high-income countries, was confirmed in this study. However, it should be noted that the loss to follow-up was significantly higher for boys, making the interpretation of the finding uncertain.

The study suggests implications for preventive actions of smoking among adolescents. The predictive value of attitudes imply that actions influencing smoking attitudes during the first six school years should be a good investment. This is consistent with the conclusion from Edvardsson and colleagues [37] stating that, in order to influence attitudes, preventive actions need to be established well before tobacco is introduced. Modelling and social reinforcement are core concepts for the understanding of factors promoting smoking [38]. Flay and colleagues [39] investigated the influence of parental and friends’ smoking on adolescents’ initiation and continuation of smoking. They conclude that parental approval of smoking, i.e. having a positive attitude, plays a significant role, more so for girls than for boys.

The strengths of the study are the longitudinal design, structured recruitment of schools with no schools refraining from participation, a moderate drop-out rate, and a relatively long follow-up time. A limitation is the higher drop-out rate among boys, adolescents born outside Sweden and snus users. Another limitation is a low statistical power to examine smoking as a predicting factor, especially in the multivariable analysis, due to the low prevalence of smoking among 12–13 year olds. This could explain why smoking in early adolescence was not significantly associated with smoking later in adolescence although the OR was high. Due to the low number of smokers it was not possible to explore subgroups of smokers (e.g. smokers divided into occasional and daily smokers and combined users). Further, the study was conducted in Sweden, a high-income country scoring high in health and social indexes, thus affecting the generalization of the results to countries with other living conditions. As noted above, a larger sample might have been able to demonstrate further significant results. One example is country of birth, where the comparatively small number of adolescents born in other countries did not allow stratification based on native country. In the multivariable analysis, important factors associated to smoking in previous studies have been included. However, some factors that previously have been found associated with adolescent smoking were not included in the questionnaire, e.g. parents smoking and smoking attitudes, and peer smoking. Furthermore, when discussing the finding regarding attitudes, it should be noted that we cannot know if the intention has varied during the elapsed time between initial assessment of the attitude, nor know what may be the causal link between attitude and subsequent behaviour. Since the beginning of this study there has been some changes in the legislation; in 2005, smoking bans in restaurants, ban of advertising outside stores selling tobacco products and ban of cigarette sales in packets with fewer than 19 cigarettes was introduced. Despite these new legislations, Swedish official data show smoking habits in upper secondary school to have been fairly stable since the end of our study in 2008 [24]. Limitations regarding the wording of the smoking question should also be noted. Adolescents’ perceptions of time, as it was defined in the question (‘these days’) might have changed between 12–13 years of age and 17–18 years of age. Hence, the possibility of time-dependent misclassification must be acknowledged. Furthermore, we lack information regarding previous smoking (information that we have regarding snus), nor was smoking status confirmed by objective measurements. However, in most studies, self-reports have been shown to be a reliable proxy for actual smoking [40].

## Conclusions

The results of this study show that in early adolescence, attitudes towards smoking, family and intrapersonal factors, gender, binge drinking, smoking and ever using snus can predict persistent smoking in later adolescence. The study stresses the importance of actions to strengthening adolescents’ self-esteem, of promoting anti-smoking attitudes in early adolescence, as well as avoidance of early initiation of snus use, implying a need of action by parents, schools, youth associations and legislating authorities.
